# 

**Published:** 1880-03

**Authors:** 


					﻿_XVb:óle Number,
CCCCXVIH.
March, 1880.
Number 3,
Vol. XL.
THE
CHICAGO
M E D I C Æ L	v
J ournal and Examiner
x	EDITORS:
N. S. DAVIS, M.D., LL.D. JAS. NEVINS HYDE, A.M., M.D.
DANIEL R. BROWER, M.D.
CHICAGO:
PUBLISHED BY THE CHICAGO MEDICAL PRESS ASSOCIATION
188 Clark Street.
‘Read th« Notice of this Journal among Advertisements.
				

